# ACORN (A Clinically-Oriented Antimicrobial Resistance Surveillance Network) II: protocol for case based antimicrobial resistance surveillance

**DOI:** 10.12688/wellcomeopenres.19210.1

**Published:** 2023-04-21

**Authors:** Yin Mo, Ying Ding, Yang Cao, Jill Hopkins, Elizabeth A. Ashley, Naomi Waithira, Prapass Wannapinij, Sue J. Lee, David L. Paterson, H. Rogier van Doorn, Paul Turner

**Affiliations:** 1 ADVANCE-ID, Saw Swee Hock School Of Public Health, National University of Singapore, Singapore, 117549, Singapore; 2 Division of Infectious Diseases, National University Hospital, Singapore, Singapore, 119074, Singapore; 3 Department of Medicine, National University of Singapore, Singapore, 119228, Singapore; 4 Centre for Tropical Medicine and Global Health, Nuffield Department of Medicine, Oxford, OX3 7BN, UK; 5 Mahidol-Oxford Tropical Medicine Research Unit, Faculty of Tropical Medicine, Mahidol University, Salaya, Nakhon Pathom, 10400, Thailand; 6 Singapore Clinical Research Institute, Singapore, 139234, Singapore; 7 Lao-Oxford-Mahosot Hospital-Wellcome Trust Research Unit, Microbiology Laboratory, Mahosot Hospital, Vientiane, Lao People's Democratic Republic; 8 Vietnam National Hospital Of Tropical Diseases, Hanoi, Vietnam; 9 Cambodia Oxford Medical Research Unit, Angkor Hospital for Children, Siem Reap, 171020, Cambodia

**Keywords:** Antimicrobial resistance, surveillance, antibiotic stewardship

## Abstract

**Background**: Antimicrobial resistance surveillance is essential for empiric antibiotic prescribing, infection prevention and control policies and to drive novel antibiotic discovery. However, most existing surveillance systems are isolate-based without supporting patient-based clinical data, and not widely implemented especially in low- and middle-income countries (LMICs).

**Methods**: A Clinically-Oriented Antimicrobial Resistance Surveillance Network (ACORN) II is a large-scale multicentre protocol which builds on the WHO Global Antimicrobial Resistance and Use Surveillance System to estimate syndromic and pathogen outcomes along with associated health economic costs. ACORN-healthcare associated infection (ACORN-HAI) is an extension study which focuses on healthcare-associated bloodstream infections and ventilator-associated pneumonia. Our main aim is to implement an efficient clinically-oriented antimicrobial resistance surveillance system, which can be incorporated as part of routine workflow in hospitals in LMICs. These surveillance systems include hospitalised patients of any age with clinically compatible acute community-acquired or healthcare-associated bacterial infection syndromes, and who were prescribed parenteral antibiotics. Diagnostic stewardship activities will be implemented to optimise microbiology culture specimen collection practices. Basic patient characteristics, clinician diagnosis, empiric treatment, infection severity and risk factors for HAI are recorded on enrolment and during 28-day follow-up. An R Shiny application can be used offline and online for merging clinical and microbiology data, and generating collated reports to inform local antibiotic stewardship and infection control policies.

**Discussion**: ACORN II is a comprehensive antimicrobial resistance surveillance activity which advocates pragmatic implementation and prioritises improving local diagnostic and antibiotic prescribing practices through patient-centred data collection. These data can be rapidly communicated to local physicians and infection prevention and control teams. Relative ease of data collection promotes sustainability and maximises participation and scalability. With ACORN-HAI as an example, ACORN II has the capacity to accommodate extensions to investigate further specific questions of interest.

## Background

Antimicrobial resistance is declared as 1 of the top 10 global public health threats facing humanity ^1^. Surveillance for the emergence and spread of antimicrobial resistance is a cornerstone of our response strategies. It is essential both at patient-level for informing empiric antibiotic prescribing and treatment guidelines, and at system-level for designing and evaluating treatment, infection prevention and control policies ^2^. Antimicrobial resistance surveillance involves systematic data collection, analysis and communication. The aim is to describe disease burden and epidemiology in a timely and unbiased manner that is relevant to the community and various stakeholders, including patients, physicians, researchers and policy makers.

Despite a global emphasis on antimicrobial resistance in recent decades, effective surveillance networks and systems remain sparse ^3^. This is mainly because assembling comprehensive surveillance data which incorporate both clinical and microbiological records is challenging. Infection episodes are defined using clinical criteria which involves sourcing individual-level data from medical notes, microbiological reports and antibiotic prescription charts. Existing population-level surveillance databases, such as the European Centre for Disease Prevention and Control Surveillance Atlas, The Center For Disease Dynamics, Economics & Policy Resistance Map, and the World Health Organisation (WHO) Global Antimicrobial Resistance and Use Surveillance System (GLASS), are isolate-based and rely mainly on microbiology data alone ^4– 6^.

These isolate-based data present geospatial and temporal trends of resistant organisms but are prone to biases introduced by local characteristics such as differential access to antibiotics in the community versus hospital settings, antibiotic prescription practices, availability of laboratory resources, and practices of microbiological culture collection and reporting. Particularly in low- and middle-income countries (LMICs), microbiology cultures may only be performed after multiple courses of empirical antibiotics have failed or in situations where diagnostics are affordable. Importantly, interpreting microbiology cultures without considering clinical syndromes makes it impossible to differentiate innocent ‘bystander’ colonising versus true pathogens, i.e. if a patient became ill or died *due to* or *with* a resistant bacteria. In addition, quantifying morbidity and mortality impacts of drug-resistant infections requires longitudinal follow-up and cannot be reliably determined from death certificates ^7^. Such high quality surveillance data is especially lacking in LMICs where antimicrobial resistance burden is the highest ^8– 10^.

A Clinically-Oriented Antimicrobial Resistance Surveillance Network (ACORN) II is a large-scale multicentre study which builds on the WHO Global Antimicrobial Resistance and Use Surveillance System (GLASS), to measure resistance by patient rather than pathogen denominators, syndromic and pathogen outcomes, along with associated health economic costs ^11^. It aims to implement an efficient clinically-oriented antimicrobial resistance surveillance system, incorporated as part of routine workflow in hospitals in LMICs. ACORN II will add value to isolate-based antimicrobial resistance surveillance, by capturing essential data on patient clinical features, management, and outcomes. In addition, ACORN II is designed to accommodate extension studies with specific focuses on infection syndrome, pathogens or patient population.

In ACORN II, data are collected from hospitalised patients of any age on designated surveillance wards in whom a parenteral antibiotic has been prescribed for treatment because of clinician suspected acute community-acquired or healthcare-associated bacterial infection. Diagnostic stewardship activities will be implemented to optimise blood culture, and syndrome specific additional culture, specimen collection in potential surveillance participants. Basic demographic characteristics, comorbidities, clinician diagnosis, empiric treatment, markers of clinical severity and risk factors for healthcare-associated infections are recorded on enrolment and during follow-up.

A pilot of ACORN was completed in 2020, which focused on three infection syndromes whilst study methodology was established and refined ^11^. In this protocol, we describe the full ACORN II study protocol, as well as the ACORN-healthcare associated infection (ACORN-HAI) extension study which focuses on healthcare-associated bloodstream infections and ventilator-associated pneumonia. Study procedures including site set up, diagnostic stewardship, data collection and rationale behind their design features are detailed below.

## Methods/design

### Ethics approval and consent to participate

An overarching approval for ACORN II and ACORN-HAI was given by the Oxford Tropical Research Ethics Committee (OxTREC, ref 524-21). Local ethics approvals are sought prior to enrolment. Written informed consent is required for participation in all study sites except the following, where anonymised data may be collected without individual consent: Angkor Hospital for Children in Siem Reap (Cambodia), Sarawak General Hospital (Malaysia), Queen Elizabeth II Hospital (Malaysia), Sabah Women and Children's Hospital (Malaysia), University Malaya Medical Centra (Malaysia), National Hospital of Tropical Diseases, Hanoi (Vietnam).

### Aims and objectives

The aim of ACORN II is to provide actionable data to local institutions, national and international surveillance systems, researchers and policy makers via an efficient clinically orientated antimicrobial resistance surveillance system (extended data 2). It is designed to be implemented alongside routine clinical care in hospitals, especially in LMIC settings. The data collected are fully compatible with WHO GLASS and are further expanded to enable classification of infection syndromes and outcomes. These data will be used to estimate syndromic and pathogen outcomes along with associated costs.

The specific objectives are to i) implement and assess a hospital-based system for patient-centred surveillance of drug resistant infections; ii) characterise drug-resistant infections by clinical syndrome, place of acquisition, patient group, and location; iii) quantify burden of drug-resistant infections in terms of attributable mortality and excess length of hospital stay (including determining the attributable mortality for extended spectrum beta-lactamase producing *Escherichia coli* and methicillin resistant *Staphylococcus aureus* bloodstream infection using the WHO attributable mortality protocol ^12^); iv) determine the major indications for prescribing parenteral antibiotics by patient group, timing of prescription, and location; and, v) to determine the major empiric antibiotics used by clinical syndrome, place of acquisition, patient group, and location.

### Study design

**Figure 1.**
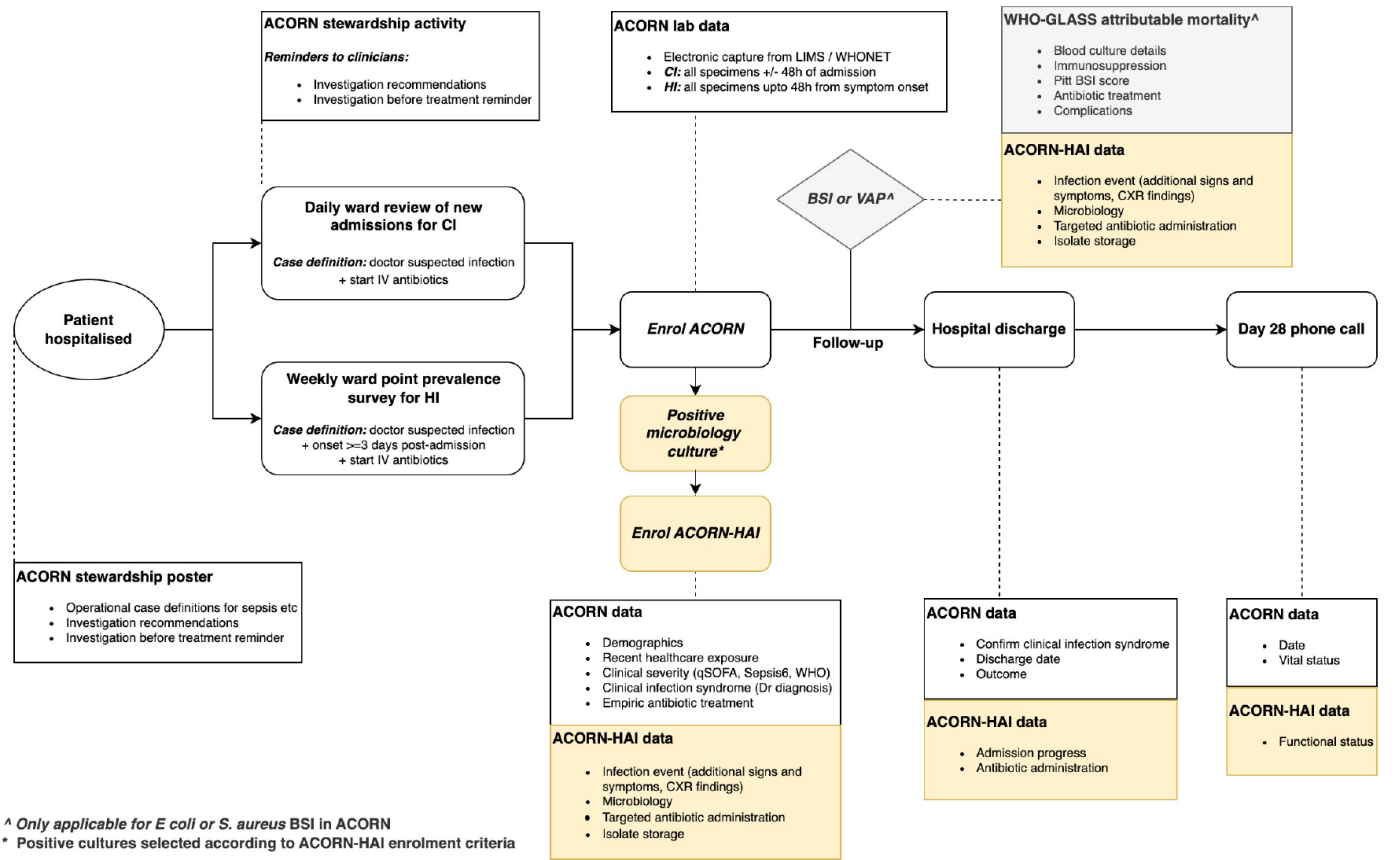
Flow diagram of key study processes for an enrolled patient. Boxes in white explain study processes in ACORN. Boxes in yellow represent additional activities and data collected for ACORN-HAI. Acronyms: CI – community acquired infection; HAI – healthcare-associated infection; BSI – bloodstream infection; VAP – ventilator-associated pneumonia.

ACORN II is a prospective observational cohort study of hospitalised patients with community-acquired and healthcare-associated bacterial infections ( Figure 1). In the ACORN II workflow, community-acquired infections (CI) are identified by daily review of new admissions to designated surveillance wards. Healthcare-associated infections (HAI), which refer to infections in patients who had significant healthcare exposure in the three months prior to admission (extended data 2), are initially enrolled as CI and will be re-classified as HAI on analysis. Hospital-acquired infections (HI) are identified during weekly point prevalence surveys on these wards. All patients who are receiving antibiotic treatment for these infections will be eligible for enrolment. Basic demographic characteristics, comorbidities, clinician diagnosis, empiric treatment, markers of clinical severity and risk factors for HAI are recorded on enrolment. Final clinician diagnosis, hospital discharge and day 28 outcome data (from the last infection episode of the admission) are collected prospectively. To contribute to the WHO GLASS attributable mortality study, additional clinical and treatment data are collected on patients with confirmed *E. coli* or *S. aureus* bloodstream infection.

Surveillance targets include, but are not limited to, pathogens that are usually associated with bloodstream infections included in WHO GLASS: *Streptococcus pneumoniae, Staphylococcus aureus, Salmonella* spp *., Pseudomonas aeruginosa, Neisseria meningitidis, Klebsiella pneumoniae, Haemophilus influenzae, Escherichia coli,* and *Acinetobacter* spp.

### Study sites

Participating sites will i) undergo basic research training such as “Good Clinical Practice (GCP)” ( https://globalhealthtrainingcentre.tghn.org/ich-good-clinical-practice/); ii) set up infrastructure such as computers, data collection software and laboratory capacity; iii) form communication channels between the research investigators, clinicians and laboratory teams for collaborations and data sharing; and iv) familiarise themselves with local ethics and regulatory bodies.

At least three acute admission wards will be selected for surveillance per site, which should ideally include a general adult medical ward, a general paediatric ward, and an intensive care unit. Consideration should be given to harmonisation with other surveillance activities, where possible. Senior clinicians, nurses and microbiology laboratory staff should be engaged as part of the study team, and understand the purpose and scope of ACORN II.

Online tools are available to guide the sites for study preparation and implementation. These cover areas such as laboratory assessment, diagnostic stewardship, and clinician knowledge, attitudes and practices (KAP) surveys:

- *Laboratory assessment*

The purpose of laboratory assessment is to make a baseline evaluation of the laboratories that will be used for processing samples and submitting data for antimicrobial resistance (AMR) surveillance. Laboratory assessments will be performed using the Laboratory Pre-Assessment online survey ( https://tinyurl.com/ACORNlabsurvey), the Site Laboratory Assessment tool (extended data 3), document review and virtual audits. The assessment tools allow the assessors to gather information on the current capacity of site laboratories including what routine specimen processing the laboratory already carries out, the current level of quality management and quality control in the laboratory, safety procedures and specimen reporting. Following completion of the laboratory assessment continual laboratory quality monitoring is performed using the Site Laboratory Quality Monitoring tool (extended data 3).

- *Assessment / implementation of diagnostic stewardship*

Diagnostic stewardship is designed to improve appropriate microbiologic testing of patients with suspected bacterial infection at ACORN II surveillance sites (extended data 3). The endpoint of diagnostic stewardship is to ensure that the right patients have the right tests at the right time and that results are used to ensure that they receive the right treatment. Systematic testing of patients with suspected infection will result in data that can be used to inform local treatment guidelines as well as be used for AMR surveillance activities. It is important that ACORN II stewardship is aligned with existing specimen collection, processing, and feedback procedures. A diagnostic stewardship checklist is available to determine the extent of existing diagnostic stewardship activities / materials at the site. Negative answers to any of the questions should prompt development of that particular item / activity in the form of recommendations for standardised investigations for suspected infection, at least covering when to collect a blood culture and / or sterile site fluid culture. Examples of appropriate guidelines are included in the ACORN diagnostic stewardship guideline and can be adapted to the local situation. Monitoring of culture rates, by patient group, clinical location, clinical syndrome, via the ACORN project app described will permit assessment of diagnostic stewardship activity success.

- *Clinician Knowledge, Attitudes and Practices survey*

The purpose of the clinician knowledge, attitudes and practices survey is to make a baseline assessment of the clinical staff working on ACORN II surveillance wards (extended data 3, https://tinyurl.com/ACORNclinicianKAP). The survey gathers information on current knowledge, attitudes and practices around diagnostic microbiology and AMR surveillance. The data will be used primarily to inform and iterate site-specific surveillance training and implementation.

### Study participants

We adopt a pragmatic approach to identify potential patients for enrolment. Patients will be enrolled based on clinician diagnosis or, in the absence of clear clinician diagnosis, if they are assessed by the surveillance team as meeting the clinical case criteria for respective infections. For CI cases, newly admitted patients will need to be identified, e.g. from a ward admission logbook. The CI inclusion criteria are:

- Patient with clinically suspected infection on admission to a surveillance ward (including those transferred directly from another facility), in whom the decision to start intravenous antibiotic treatment has been made, and are willing to participate in the surveillance. This includes ^1^:
  - Patients transferred directly from another facility with an acute infection;
  - Patients admitted to a non-surveillance ward initially but transferred to a surveillance ward within 48 hours of admission;
  - Patients investigated and treated for suspected CI in the Emergency Department (Emergency Room / Accident and Emergency Department) with delayed transfer to the surveillance ward for any reasons, e.g. bed shortages, COVID-19 screening procedures, or other local operational challenges.

For HI cases, all admitted patients in the surveillance wards will be reviewed on a single day per week via a point prevalence survey (PPS). The HI inclusion criteria include:

- Clinical suspicion of bacterial infection and prescription / commencement of a new intravenous antibiotic (but not escalation of antibiotic treatment for an existing suspected or proven infection); and
- Onset of infection syndrome on or after day 3 of admission (day 1 refers to the day of admission); and
- Infection syndrome was not active during the previous weekly review, i.e. onset at least one day following the most recent previous healthcare-associated infection point prevalence survey

An overarching approval for ACORN II to enrol without the need for individual consent was given by the Oxford Tropical Research Ethics Committee (OxTREC, ref 524-21). The requirement for informed consent will be catered to local ethics committees’ advice. In participating sites where the need for explicit informed consent can be waived for minimal / negligible risk studies, potential participants in participating wards will be given a patient information sheet with details about the study. At least one ACORN II information poster will be on display in each surveillance ward. Potential study participants will be asked to confirm verbally that they (or legally acceptable representatives) have read the surveillance information material and agree to participate. Any patient who requests not to be included in the study will be recorded accordingly in the screening logbook. In sites where informed consent cannot be waived, a typical consent taking process will be in place.

Further infection episodes may be identified during the weekly PPS. The final follow up is 28 days after the final infection episode. This can be done by telephone if the participant has been discharged. If the patient is readmitted before the 28 day follow up, the 28 day follow up should be completed as scheduled if no further infection episodes are diagnosed during the readmission.

### Data collection and management

**Figure 2.**
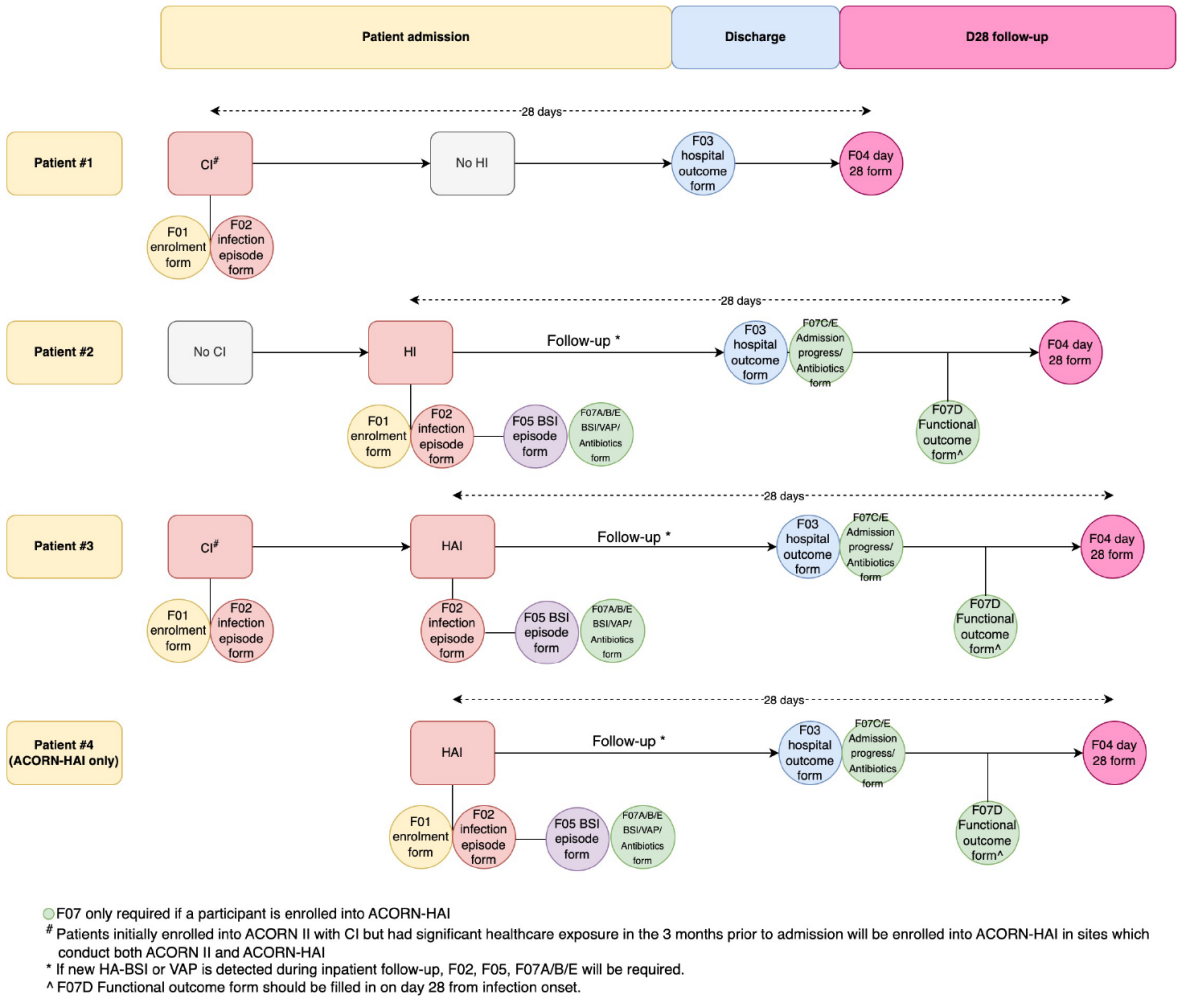
Data collection procedures during enrolment and follow-ups. Patient 1 represents a participant who enrolls into ACORN II with a community-acquired infection diagnosed on day 1 of hospitalisation. Patient 2 represents a participant who enrols into both ACORN II and ACORN-HAI with a hospital-acquired infection which developed after 2 days of hospitalisation. Patient 3 represents a participant who enrolls into both ACORN II initially with a community-acquired infection, and then enrolls into ACORN-HAI after developing a hospital-acquired infection after 2 days of hospitalisation. Patient 4 represents a participant who enrolls into ACORN-HAI (from a study site which only implements ACORN-HAI) with a hospital-acquired infection after 2 days of admission.

On enrolment, baseline clinical data will be extracted from the patient clinical records/electronic hospital information systems ( Figure 2):

- Demographics
- Date of admission and ward location
- Primary reason for admission
- Co-morbidity status (modified Charlson comorbidity index)
- Healthcare exposure in the three months before current admission

The following data will be collected about the infection episode on enrolment and when a new infection episode is detected in the weekly ward PPS:

- Ward details
- Infection syndrome

- Clinical severity signs at symptom onset
  - qSOFA score for adults, ≥18 years ^13^
  - Sepsis six recognition features for children, <18 years ^14^
  - General WHO severity signs for neonates, <28 days

- Presence of medical devices / surgical procedures
- Microbiology and antibiotic susceptibility
- Empiric antibiotic treatment prescribed

For patients with laboratory proven *E. coli* or *S. aureus* BSI, additional clinical data are captured to fulfil the requirements of the WHO GLASS attributable mortality protocol ^12^. The Pitt BSI score is calculated and details of immunosuppression are collected, along with complete details of antibiotic treatment for the infection and likely source of infection.

Clinical data entry can be done either directly using the Open Data Kit (ODK) Collect app on an Android tablet or via paper case report forms (CRFs) with subsequent entry into the surveillance REDCap database using a laptop and web browser. All clinical data entered will be stored centrally in the ACORN REDCap database, where error checking and correction is performed regularly. All data stored in the central database are anonymised and securely stored.

**Figure 3.**
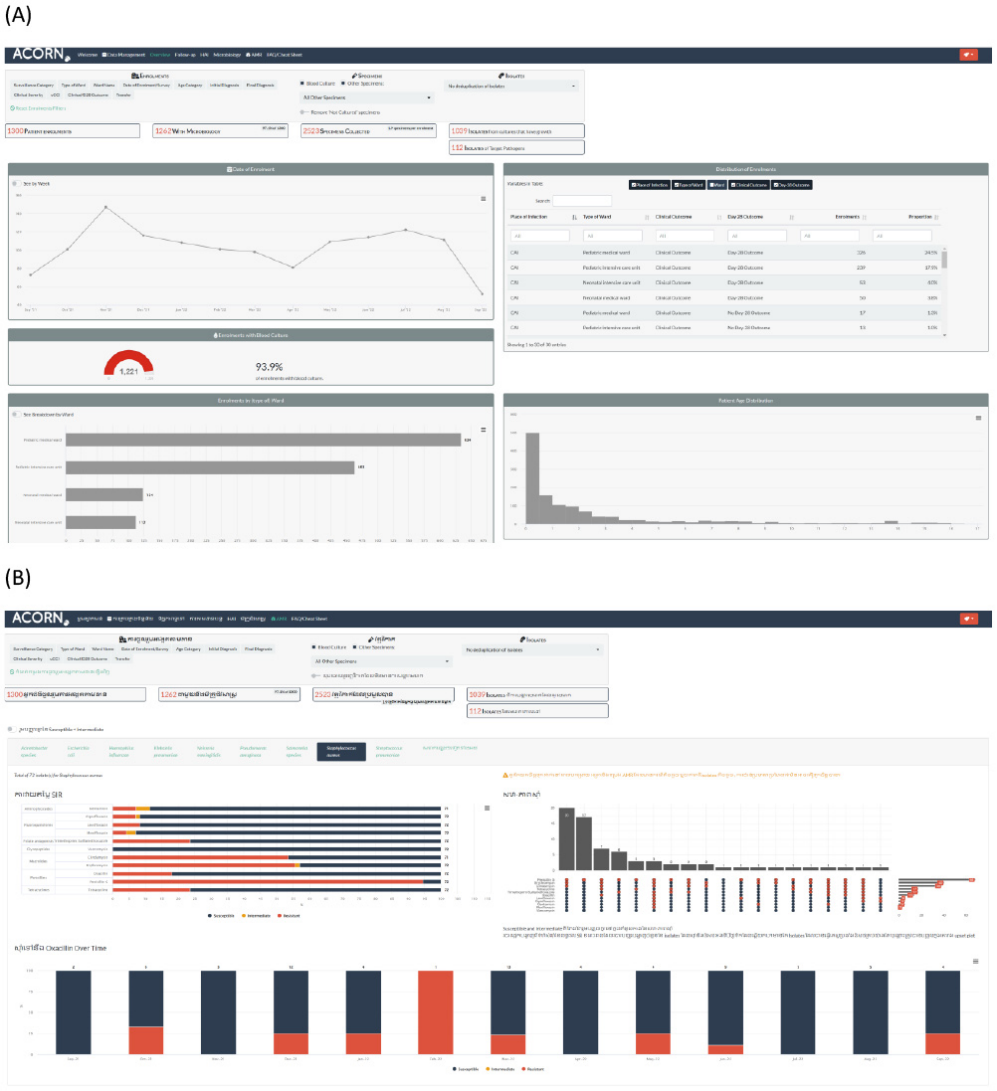
The ACORN project app / dashboard. Panel **A** shows part of the overview tab highlighting the data filters and high-level metrics. Panel **B** (Khmer language version) shows the AMR tab with data for *S. aureus*: standard SIR proportion plot (left side), an UpSet plot to summarise co-resistance patterns (right side), and temporal trends for key antimicrobials (methicillin, bottom).

Microbiology laboratory data, either extracted from an existing laboratory information management system (LIMS) / WHONET file or entered into WHONET software specifically for surveillance, is linked to clinical data using the ACORN project RShiny application ( https://github.com/acornamr/acorn-dashboard) ( Figure 3). This data management application links clinical and laboratory data automatically based on patient identifier and key dates. To ensure data privacy and security, this application can be used offline to link laboratory data and pseudonymise the dataset. An identical online version is also available ( https://moru.shinyapps.io/acornamr/). The application allows for real-time monitoring of surveillance and can be used for data visualisation and reporting. If it is not possible to access existing laboratory LIMS or WHONET data extracts, microbiology data can be directly entered into a WHONET database specifically for the project.

### ACORN-HAI extension study

ACORN-HAI follows the ACORN II main study methodologies and procedures closely, with a specific focus on healthcare-associated bloodstream infection and ventilator-associated pneumonia (extended data 4 and 5). These two infection syndromes are chosen due to their association with drug resistant organisms and high mortality. In ACORN-HAI, healthcare-associated bloodstream infections encompass hospital-acquired bloodstream infections, i.e. any bloodstream infections taking place either after two days of admission or within 3 months of significant healthcare exposures, can be enrolled into the study.

A participating site may participate in both or either ACORN II and ACORN-HAI study. In sites which implement both ACORN II and ACORN-HAI, only study participants who are enrolled into ACORN II will be enrolled into ACORN-HAI. Additional variables required by ACORN-HAI will be collected with an addendum CRF ( Figure 2). In sites or wards which participate only in ACORN-HAI, similar clinical, microbiology and ward-level data as the ACORN II study will be collected such that the two datasets can be merged and analysed in a coherent manner ( Table 1). Rather than using the weekly point-prevalence surveys, in sites that only participate in ACORN-HAI, enrolment is triggered by having a positive culture result.

**Table 1.** Comparison of main ACORN II and ACORN-HAI activities.

Activity	ACORN II	ACORN-HAI
Screening	◦ Community-acquired infections via daily ward reviews (which can include healthcare-associated infections among patient with significant healthcare exposure in 3 months prior to admission) ◦ Hospital-acquired infections via weekly point prevalence surveys	Healthcare-associated infections (which include hospital-acquired infections) via microbiology line lists
Data collection for specific infection syndromes	Various types of community- and hospital- acquired infection syndromes (detailed data collection for *E. coli* and *S. aureus* bloodstream infections)	Culture-positive healthcare- associated bloodstream infections and ventilator-associated pneumonia
Follow-up	During weekly point prevalence surveys (for hospital-acquired infections) and at discharge	Weekly during admission and at discharge
Pathogen isolate storage	No pathogen isolate storage required	Storage of pathogen isolates associated with infection episodes
Outcome assessment	Vital status	Vital and functional status

Enrolment criteria for ACORN-HAI are as follows:

- *Bloodstream infection*

Inclusion criteria:

1) Prescription/commencement of an intravenous antibiotic;

2) Growth of bacterial or *Candida* spp *.* pathogen(s) identified from blood specimen(s) ^2^ either taken on or after day 3 of admission (Day 1 refers to the day of admission), or within 3 months of significant healthcare exposures ^3^; and 

3) Pathogen(s) in the blood specimen(s) satisfies either of the following:

*i.*  1 or more non-common commensal ^4^ bacterial or *Candida* spp. pathogen(s) identified from 1 or more blood specimens obtained by a culture; or
*ii.*  the same common commensal bacterial pathogen identified from 2 or more blood specimens collected on separate occasions ^5^; and

4) The same pathogen(s) in the blood specimen(s) was/were not present in the blood specimen(s) taken during the first 2 days of admission among those without significant healthcare exposures in the past 3 months.

Exclusion criteria:

Patient who has positive growth of organisms belonging to the following genera which are typically causes of community-associated infections and are rarely or are not known to be causes of healthcare-associated infections, or associated with severe immune suppression:

a) *Burkholderia pseudomallei*,
b) *Brucella* spp., including but not limited to, *B. melitensis, B. abortus, B. suis, B. canis,*
c) *Campylobacter, Salmonella, Shigella, Listeria, Vibrio and Yersinia.*

- Ventilator-associated pneumonia

1) Prescription/commencement of an intravenous antibiotic;

2) Clinical suspicion of ventilator-associated pnuemonia ^6^; and 

3) Growth of bacterial pathogen(s) identified from respiratory specimen(s) ^7^ taken on or after day 3 of ventilation.

Exclusion criteria:

Patient who has positive growth of organisms belonging to the following genera which are typically causes of community-associated infections and / or are rarely or are not known to be causes of healthcare-associated infections:

a) *Streptococcus pneumoniae, Streptococcus suis, Haemophilus influenzae,*
b) *Burkholderia pseudomallei*,
c) Coagulase-negative staphylococci,
d) *Talaromyces marneffei,*
e) *Mycobacterium tuberculosis,*
f) Rapidly growing Mycobacteria, including but not limited to, *M. mucogenicum, M. fortuitum, M. abscessus, M. chelonae, M. neoaurum*

Bacterial isolates including *Acinetobacter* spp., *Pseudomonas* spp., Enterobacterales, *Staphylococcus aureus*, *Enterococcus* spp. and Candida (only from positive blood cultures), grown from routine clinical cultures will be collected from the microbiology laboratory and stored. The isolates will undergo whole genome sequencing at accredited laboratories.

### Sample size calculation

There is no formal sample size calculation. This surveillance will enrol all eligible and consenting patients admitted to the surveillance wards during the surveillance period.

### Study monitoring and quality assurance

All participating sites will develop an internal data monitoring plan to ensure adherence to ethics requirements, completeness and accuracy of data entry, and if applicable, isolate collection and storage. The local and overall data manager will conduct regular audits of the data entered and raise queries when necessary. Collated reports for monitoring progress and quality of data collected will be generated and presented to participating sites regularly.

### Surveillance data analysis and communication

ACORN II site teams will interact with their local data in real time using the project app / dashboard. Via this dashboard, site investigators will have access to enrolment frequencies and patient demographics, follow-up on clinical outcome and day 28 status, weekly active HI point prevalence, microbiology summaries, and antimicrobial susceptibility and resistance patterns.

The secondary outcomes are to: i) characterise multidrug resistant infections by clinical syndrome, place of acquisition (CI, HAI/HI), patient age group (adult, paediatric, neonatal), sample type, and location (site, country, region), ii) quantify burden of multidrug-resistant infections in terms of attributable mortality and excess length of hospital stay, and iii) determine major parenteral antibiotic prescription indications by clinical syndrome, patient group (adult, paediatric, neonatal), timing of prescription (empirical versus definitive), and location (site, country, region). Analyses for these secondary outcomes will be performed after at least two years from study commencement (extended data 6).

In general, data will be summarised in tables and graphs using descriptive statistics. For each selected key pathogen, the proportions of cases will be calculated, using the total number of participants from whom any pathogen was isolated as the denominator. Summaries will include the proportions of isolates resistant to key antibiotics, as defined by WHO GLASS or categorised as multidrug resistant, using standard definitions. Univariable and multivariable logistic regression models will be fitted to explore whether any clinical or microbiological variables are associated with the outcomes of resistance, mortality and discharged moribund. The unit of analysis will be admissions, with patient and site fitted as random effects.

For attributable mortality analyses, the survival model approach outlined in the WHO GLASS protocol will be followed ^15^. At each site, observed crude case fatality rates (survival data) will be compared between cohorts: patients with multidrug resistant infection for selected pathogen-antimicrobial combination (cohort 1), and patient with non-multidrug resistant infection for selected pathogen-antimicrobial combination (cohort 2). Patients from cohort 1 and 2 will be matched 1:1 retrospectively. Ideally, matching will be by age category, admission ward, month of infection, and clinical syndrome at enrolment, and by the time from admission to infection. The effect of antibiotic resistance on vital status will be estimated using cause-specific Cox proportional hazards models, assessing the competing events of mortality and discharge alive, from the time of infection. Admissions, rather than patients, will be used as the unit of analysis and only the first relevant infection episode will be considered. A composite all-cause end- of-stay endpoint (either death or discharge alive) will also be assessed which may be interpreted as an indication of the daily hazard of a patient’s admission ending. All models will include adjustment for the time between admission and infection. Proportional hazards assumptions will be checked using Schoenfeld residuals and visual inspection of log-log plots. Non-proportional hazards will be corrected using stratification.

Excess length of stay in days will be calculated using multistate models. The difference in expected length of stay will be estimated between the multidrug resistant and susceptible states. For empiric antibiotic analyses, drugs prescribed on the day of admission or symptom onset will be classified according to the WHO AWaRe criteria ^9^. Concordance (i.e. cultured isolate was susceptible) or discordance (i.e. cultured isolate was resistant) with microbiology test results will be determined. To assess the impact of initial treatment on mortality another set of Cox proportional hazards models will be run, but antibiotic resistance will be replaced by receipt of active initial therapy as the exposure of interest.

To examine trends in infection rates over time, we will first use plots to visually inspect the trends to determine the most appropriate model/approach for analysis, while anticipating that random effects modelling with hospital clusters will be likely. Monthly incidence will be modeled (i.e., cases per hospital-month) controlling for hospital characteristics including the country’s income status, type of hospital (tertiary versus community), and proportion of patients in specific age ranges ^16^.

In the ACORN-HAI study, all clinical isolates will be processed for genomic sequencing. These genomic data will inform geospatial distribution of pathogen species and phenotypic resistance patterns and trends over the study period.

## Study status

**Figure 4.**
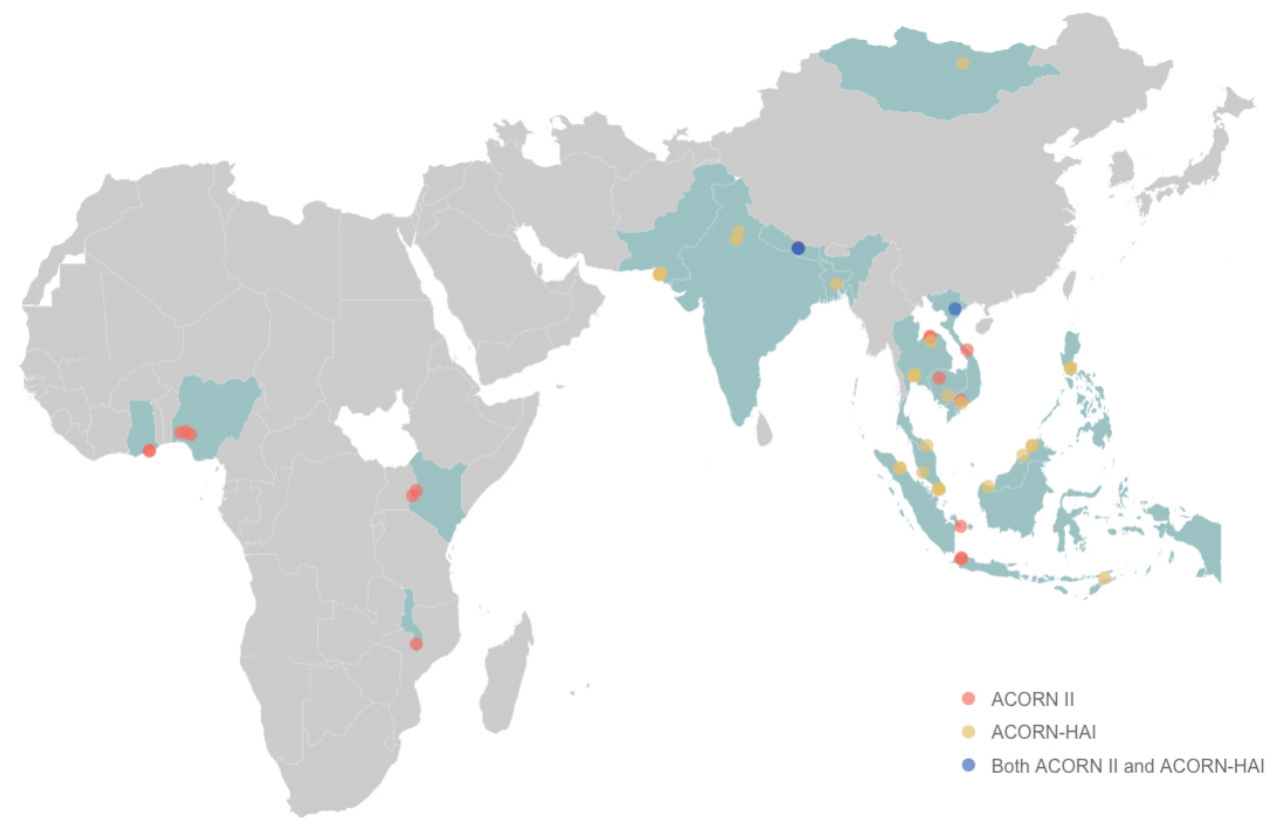
ACORN II and ACORN-HAI participating countries and study sites. Participating countries are highlighted in light teal. Study sites are represented by diamond points: ACORN II sites (coral), ACORN-HAI sites (yellow), ACORN II and ACORN-HAI sites (blue).

ACORN II commenced in September 2021 in Asia and Africa and is actively enrolling in 19 hospitals. The first study site participating in ACORN-HAI started enrolment in September 2022 and will be implemented in 30 hospitals across Asia including Singapore, Malaysia, Vietnam, Thailand, Nepal, India, and Pakistan. These hospitals include university academic and provincial-level centres ( Figure 4). 

## Discussion

ACORN II is a comprehensive antimicrobial resistance surveillance activity which advocates pragmatic implementation, and prioritises improving local diagnostic and antibiotic prescribing practices through patient-centred data collection and rapid feedback to physicians. It uses a systematic methodology to collect clinical, microbiology, and antibiotic use data. These data can be rapidly communicated to local physicians and infection prevention and control teams via an interactive web application to guide empiric antibiotic prescription and stewardship. Relative ease of data collection promotes sustainability and maximise participation and scalability.

A prospective cohort design has strengths with regards to less risk of bias and confounding when compared to cross-sectional and retrospective study designs, albeit at the expense of higher cost. Important confounders in evaluating burden of drug resistant infections, e.g. attributable mortality, include comorbidities, initial disease severity at symptom onset, prior antibiotic exposure and time from admission to infection ^17^. In addition, prospectively collected data is less prone to information bias as relevant data are collected at baseline (without knowledge on outcomes) using standardised methods ^17^. Selection bias is minimised by implementing this surveillance tool in multiple antimicrobial resistance ‘hotspot’ regions to achieve a representative cohort of patients with multidrug-resistant infections.

ACORN II has the potential to fill important gaps in our understanding of antimicrobial resistance epidemiology. Firstly, most existing antimicrobial resistance surveillance systems are passive, and based on routine antimicrobial susceptibility testing results generated by clinical microbiology laboratories alone. While such designs are useful to give broad pictures on the types of resistance and bacteria prevalent in a population, they cannot be used to relate the effect of resistance with patient outcomes. These proportions of resistance per pathogen antimicrobial combination lack relevant clinical metadata to be informative for local clinicians in their decision making or guideline development. Secondly, ACORN II focuses on LMICs where there is a mismatch in terms of antimicrobial resistance burden and surveillance capacity. ACORN II presents opportunities for participating sites to adopt and integrate a systematic surveillance programme into their routine workflows.

By focusing on severe healthcare-associated infections, ACORN-HAI builds on the main ACORN II protocol by collecting more detailed data on disease presentation, progression and treatment. The commonest pathogens causing healthcare-associated bloodstream infections and ventilator-associated pneumonia in LMICs are carbapenem-resistant *Acinetobacter baumannii*, carbapenem-resistant *Pseudomonas aeruginosa*, carbapenem-resistant Enterobacterales and extended-spectrum beta-lactamase-producing Gram-negative bacteria ^18– 20^. These antibiotic-resistant pathogens have been identified by the WHO as of the highest concern ^21^. This is because treatment options for carbapenem-resistant organisms are severely limited, associated with high toxicity, and often ineffective. ACORN-HAI, led by the ADVANCE-ID (Advancing Clinical Evidence for Infectious Diseases) network, will form the groundwork for subsequent interventional clinical trials targeting drug resistant infections. This reflects ADVANCE-ID’s main mission, which is to promote transnational research collaboration for a sustainable pipeline to conduct large-scale clinical trials in infectious diseases, particularly tackling antimicrobial resistance in LMICs where the need is the most urgent. With ACORN-HAI as an example, we foresee such extensions based on the ACORN II protocol in the future as hospital and research networks identify specific questions of interest.

Implementation of a robust surveillance network for drug resistant infections is the necessary first step towards improved empirical antibiotic prescription, designing infection prevention and control policies, guiding resource allocation, and motivating novel treatment therapy clinical trials. A focus on severe drug resistant infections with the highest health and economic consequences will align the interests and expectations from various stakeholders including patients, clinicians, health ministries and pharmaceutical industry. Such surveillance data will engage all parties to direct a concerted effort to tackle the urgent issues in antimicrobial resistance. 

## Data Availability

No data are associated with this article. Zenodo: ACORN (A Clinically-Oriented Antimicrobial Resistance Surveillance Network) II: protocol for case based antimicrobial resistance surveillance. https://doi.org/10.5281/zenodo.7823119 ^22^ This project contains the following extended data: - 1_investigatorslist.docx - 2_ACORN2 protocol.docx - 3_ACORN2CRF.pdf - 4_ACORNHAIprotocol.docx - 5_ACORNHAICRF.docx - 6_ACORN2SAP.docx Data are available under the terms of the Creative Commons Attribution 4.0 International license (CC-BY 4.0).

## Footnotes

^1^ CI patients may be reclassified as HAI if risk factors for HAI are present at enrolment.

^2^ Catheter tip cultures cannot be used in place of blood specimens for inclusion criteria.

^3^ Significant healthcare exposures refer to any of the following: admission to other healthcare facilities, regular contact with healthcare facilities (e.g. dialysis, outpatient antibiotic or chemotherapy), surgical procedures.

^4^ Common commensal organisms include, but are not limited to, diphtheroids ( *Corynebacterium* spp. not *C. diphtheriae*), *Bacillus* spp. (not *B. anthracis*), *Propionibacterium* spp., coagulase-negative staphylococci (including *S. epidermidis*), viridans group streptococci, *Aerococcus* spp., *Micrococcus* spp. and *Rhodococcus* spp.

^5^ 2 or more blood specimens collected on separate occasions are defined by samples collected on: the same calendar days from 2 separate sites (e.g. different venepunctures, different lumens of the same central catheter); or, different calendar days from the same or separate sites.

^6^ Mechanical ventilation refers to any device used to support, assist or control respiration (inclusive of the weaning period) through the application of positive pressure to the airway when delivered via an artificial airway, specifically an oral/nasal endotracheal or tracheostomy tube. Ventilation and lung expansion devices that deliver positive pressure to the airway (for example: CPAP, Bipap, bi-level, IPPB and PEEP) via non-invasive means (for example: nasal prongs, nasal mask, full face mask, total mask, etc.) are not considered ventilators unless positive pressure is delivered via an artificial airway (oral/nasal endotracheal or tracheostomy tube).

^7^ Respiratory tract specimens refer to endotracheal aspirate, bronchoalveolar lavage or protected specimen brushing. Other methods of specimen collection, e.g., cheek or mouth swabs and endotracheal tube cultures, are not acceptable.
