# Supplementary material for: ACORN (A Clinically-Oriented Antimicrobial Resistance Surveillance Network) II: protocol for case based antimicrobial resistance surveillance
Source: Wellcome Open Res. 2023 Aug 16;8:179. Originally published 2023 Apr 21. [Version 2] doi: 10.12688/wellcomeopenres.19210.2 (PMC10579854; doi:10.12688/wellcomeopenres.19210.2)
Supplement: Peer review history

# Reviewer report: Reviewer response for version 2

**DOI:** 10.21956/wellcomeopenres.21733.r65384

**Published:** 2023-10-16

**Authors:** Bashar Haruna Gulumbe

**Affiliations:** 1 Federal University Birnin Kebbi, Birnin Kebbi, Kebbi, Nigeria

In response to my prior feedback, the authors have provided thoughtful revisions to the manuscript. I appreciate their clarification on the evolution of the ACORN study, detailing the transition from ACORN I (pilot) to ACORN II, and further to the extension study, ACORN-HAI. Their emphasis on the synergy between ACORN II and ACORN-HAI, especially in the context of enrolling an increased number of patients with HAI and capturing in-depth clinical data, is well-articulated. Furthermore, the inclusion of key study limitations in the discussion adds depth and transparency to the paper. The improvements notably address the initial concerns and enhance the manuscript's overall clarity and contribution.

Is the study design appropriate for the research question?

Partly

Is the rationale for, and objectives of, the study clearly described?

Yes

Are sufficient details of the methods provided to allow replication by others?

Partly

Are the datasets clearly presented in a useable and accessible format?

Not applicable

Reviewer Expertise:

Microbiology,  AMR, infectious diseases

I confirm that I have read this submission and believe that I have an appropriate level of expertise to confirm that it is of an acceptable scientific standard.

# Reviewer report: Reviewer response for version 2

**DOI:** 10.21956/wellcomeopenres.21733.r65383

**Published:** 2023-10-16

**Authors:** Dickson Aruhomukama

**Affiliations:** 1 Makerere University, Kampala, Central Region, Uganda

I have thoroughly reviewed the current version provided by the authors, and I am pleased to note that the majority of my concerns have been effectively addressed. I extend my appreciation to the authors for their diligent efforts and commendable work. I wish them all the best moving forward.

Is the study design appropriate for the research question?

Partly

Is the rationale for, and objectives of, the study clearly described?

Partly

Are sufficient details of the methods provided to allow replication by others?

Partly

Are the datasets clearly presented in a useable and accessible format?

Not applicable

Reviewer Expertise:

Clinical Microbiology, Computational Microbiology,  Clinical Bioinformatics (Genomics)

I confirm that I have read this submission and believe that I have an appropriate level of expertise to confirm that it is of an acceptable scientific standard.

# Reviewer report: Reviewer response for version 1

**DOI:** 10.21956/wellcomeopenres.21292.r58454

**Published:** 2023-06-19

**Authors:** Bashar Haruna Gulumbe

**Affiliations:** 1 Federal University Birnin Kebbi, Birnin Kebbi, Kebbi, Nigeria

The article presents the protocol for ACORN II, a surveillance network aimed at monitoring antimicrobial resistance (AMR) in a clinically oriented manner. The study focuses on patients with severe bacterial infections in low- and middle-income countries (LMICs), where the burden of AMR is high and surveillance capacity is limited.

 The ACORN II surveillance network aims to provide a comprehensive picture of AMR, focusing on both community-acquired and healthcare-associated infections. The study includes patients with clinically compatible acute bacterial infection syndromes and collects data on patient characteristics, infection syndromes, microbiology, and antibiotic use. The data is then rapidly communicated to local physicians and infection prevention and control teams via an interactive web application to guide empiric antibiotic prescription and stewardship.

 The study also includes an extension, ACORN-HAI, which focuses on healthcare-associated bloodstream infection and ventilator-associated pneumonia due to their association with drug-resistant organisms and high mortality. The ACORN-HAI study follows similar methodologies and procedures as the main ACORN II study but collects more detailed data on disease presentation, progression, and treatment.

 Constructive Feedback:

 Clarify the Objectives/methods: The authors should provide a clearer link between the specific objectives of the study and the methods used to achieve them. This would help readers understand how the study design and data collection methods will help achieve the stated objectives.

 Clarify the Role of ACORN-HAI: The authors introduce ACORN-HAI as an extension of ACORN II, but it's not clear how these two studies are related. The authors should provide more information on the role of ACORN-HAI and how it fits into the overall ACORN II study.

 Limitations: Every study has limitations, and it's important for the authors to acknowledge these. For instance, are there potential biases in the study design? How generalizable are the findings likely to be, given that the study is being conducted in LMICs?

 Figures: The content of Figures 3A and 3B is not easily readable, which limits their usefulness in the context of the study.

 Overall, the article presents a valuable protocol for monitoring antimicrobial resistance in LMICs. With some improvements in clarity and detail, it could serve as a useful guide for other researchers in this field.

Is the study design appropriate for the research question?

Partly

Is the rationale for, and objectives of, the study clearly described?

Yes

Are sufficient details of the methods provided to allow replication by others?

Partly

Are the datasets clearly presented in a useable and accessible format?

Not applicable

Reviewer Expertise:

Microbiology,  AMR, infectious diseases

I confirm that I have read this submission and believe that I have an appropriate level of expertise to confirm that it is of an acceptable scientific standard, however I have significant reservations, as outlined above.

# Reviewer report: Reviewer response for version 1

**DOI:** 10.21956/wellcomeopenres.21292.r58460

**Published:** 2023-06-01

**Authors:** Dickson Aruhomukama

**Affiliations:** 1 Makerere University, Kampala, Central Region, Uganda

I applaud the team for their brilliant work. The authors' aim to incorporate patient- or clinically-oriented AMR surveillance alongside isolate-based AMR surveillance is important because it acknowledges the multifaceted nature of AMR and can enable a comprehensive understanding of the problem. This approach can enhance the development of effective strategies to combat AMR and promote better patient outcomes through targeted interventions and improved antimicrobial stewardship. Upon evaluating this protocol, I have the following observations:

 The authors describe ACORN-II and ACORN-HAI in this protocol. In the title, they present this as an ACORN-II protocol. They describe a surveillance system that includes hospitalized patients… with clinically compatible acute community-acquired or healthcare associated bacterial infection syndromes… They also mention diagnostic activities that will be implemented to optimize culture specimen collection practices and later state that patient characteristics are already being recorded on enrolment and after 28 days of follow up...

 My concern with the authors’ approach is that they mix various elements in this protocol, making it somewhat unclear. Moreover, the fact that the protocol is already being implemented adds complexity to evaluating its suitability.

 I would have preferred the authors to follow a more systematic flow in their writing. They should have summarized ACORN-I since it presumably serves as the baseline for ACORN-II. Additionally, they should have described the advancements or updates in ACORN-II compared to ACORN-I, such as novel approaches, methodologies, or updated protocols etc. For instance, ACORN-I (the pilot study) focused on three infection syndromes, and it would be helpful to clarify which of these ACORN-II focuses on and why?

 Currently, the authors seem to present ACORN-I as GLASS, which is somewhat perplexing. Since they state that ACORN-II builds on the WHO-GLASS, to measure... I think they need to base ACORN-II more on the pilot study (ACORN-I) and less on GLASS.

 The authors briefly mention a surveillance system but fail to provide a comprehensive description of its components. Is it unclear whether this system encompasses ACORN in general, including both ACORN-I and ACORN-II, or if it specifically refers to ACORN-II and its extensions, such as ACORN-HAI? Although the authors introduce ACORN-HAI as an extension of ACORN-II, they should consider describing it fully in this protocol since it appears to be an independent study on its own. It would be helpful for the authors to clearly distinguish between ACORN-II and ACORN-HAI and explain the rationale behind the extension (ACORN-HAI).

 The authors mention ongoing activities while also stating that some activities are not yet underway (based on their writing tenses). This makes it challenging to provide feedback on the protocol if certain study activities are already underway. I would appreciate clarification from the authors to better understand what has been done, what is in progress, and what remains to be done.

 Regarding the study design versus the specific objectives, the authors state that ACORN-II is a prospective observational study (that is already in progress). This implementation phase makes it difficult to provide comments on a protocol that is already being executed. However, I am concerned that the general design may not effectively achieve the specific objectives outlined by the authors (i to v). I recommend that the authors provide further clarification in this regard.

 Lastly, I suggest that the authors clearly define the methods that they intend to use to achieve each specific objective in a systematic manner. This would facilitate a more thorough evaluation and allow for feedback on the proposed approaches.

Is the study design appropriate for the research question?

Partly

Is the rationale for, and objectives of, the study clearly described?

Partly

Are sufficient details of the methods provided to allow replication by others?

Partly

Are the datasets clearly presented in a useable and accessible format?

Not applicable

Reviewer Expertise:

Clinical Microbiology, Computational Microbiology,  Clinical Bioinformatics (Genomics)

I confirm that I have read this submission and believe that I have an appropriate level of expertise to confirm that it is of an acceptable scientific standard, however I have significant reservations, as outlined above.
